# Withstanding as You Withdraw and Withhold Support: Silence of Our Words

**DOI:** 10.1089/jpm.2021.0177

**Published:** 2021-09-20

**Authors:** Roy K. Philip

**Affiliations:** ^1^ University Maternity Hospital Limerick (UMHL), Limerick, Ireland.; ^2^ Division of Neonatology, Department of Paediatrics, University of Limerick School of Medicine, Limerick, Ireland.

*Withstanding the vicissitudes of emotions in the midst of life-limiting decision making in neonatology is depicted in a poem. Positively embracing inaction when one is trained otherwise in this critical care subspecialty could even be regarded by some as counterintuitive. At 34 weeks of gestation with rapidly increasing head circumference and antenatal MRI confirming inoperable severe hydrocephalus with 3mm mantle of cerebral cortex, a collective decision was reached along with parents to deliver the infant and not to offer interventions. Ensuring the pre-agreed palliative and end-of-life care-plan, while supporting the emotional burden of parents and staff is often challenging*.

Tears in her left eye said, thank you

Spilling over to the cheek, said thanks again.

Tears in her right eye said, sorry

Not to me, to her son for whom I did nothing.

He cried swiftly at birth, I opted not to hear

He sneezed twice or thrice, I chose not to assist

He cried again a bit louder, as if I didn't hear it first

He looked at me once; I gazed away like a coward.

Withdrawing is tough, withholding is tougher

Justifying my actions, in the midst of muddled thoughts

May be this is tough love, if so in its purest way.

May be this is destiny, if so I'm just a catalyst.

I gently closed his eyes, my only good deed of the day

His thin mantle of brain whispered, thank you.

Now I'm confused, has he a mind?

Was it the life, mind or soul that left him today?

Perhaps I played a part, just a plea for my passivity

Escorted another life away, to a world unknown to me

Pardon me, pardon me, I said in silence

I'm sure he heard it well, though no one else could.

